# Supplementary material for: Two Retrotransposon Elements in Intron of Porcine BMPR1B Is Associated with Phenotypic Variation
Source: Life (Basel). 2022 Oct 20;12(10):1650. doi: 10.3390/life12101650 (PMC9604734; doi:10.3390/life12101650)
Supplement: Supplementary file 1 [file life-12-01650-s001.zip › life-1919599-supplementary.pdf]

life

MDPI

Supplementary material of Two Retrotransposon Elements in Intron of Porcine *BMPR1B* Is Associated with Phenotypic Variation

Table S1: Number and origin of pig breeds for *BMPR1B* RIPs detection

Breed	Number	Province/country of origin
Large white	556	Anhui/China
Duroc	24	Anhui/China
Landrace	24	Anhui/China
Sujiang	21	Jiangsu/China
Sushan	32	Jiangsu/China
Fengjing	24	Jiangsu/China
Meishan	24	Jiangsu/China
Erhualian	24	Jiangsu/China
Wuzhishan	24	Hainan/China
Bama	6	Jiangsu/China
Diannan small-ear	6	Yunnan/China
Tibetan	6	Jiangsu/China
Wild boars	6	Anhui/China

Table S2: The primers for PCR, vectors construction and q-PCR

Primer name	Forward primer	Reverse primer	Purpose
BMPR1B-SINE-RIP1	F: TCCATCTCAGCCATAGCAAC	R: GTGCGCTCATTTATAAGGCAT	RIPs identification
BMPR1B-SINE-RIP2	F: AACAGATGGCTCCTATCATCC	R: ATTCCTTCCTATGACCCGTTC	
BMPR1B-SINE-RIP3	F: AAGAAGCCCAGGAGATCCAA	R: CCTCAGGCTCATTGTGTCGAA	
BMPR1B-SINE-RIP4	F: GAACATACAGCTCCAGTGACC	R: GCTGCTATTCTTCAGATACGTT	
BMPR1B-SINE-RIP5	F: GTCCTCTTACAGAATTTGCCAT	R: AACTGCAGCTGTGAACTCA	
BMPR1B-SINE-RIP6	F: CCTAATTCGCAAAGATTTTCATAACACT	R: AAAATGATCTGTGCTTATTGTAGAGGT	
BMPR1B-SINE-RIP7	F: CTGAGCCTAGAACTTTAACCC	R: TTCTTCATTCCCACGTAGCTT	
BMPR1B-ERV-RIP8	F: TGTGTTAAAACTATTGACCCTC	R: GCACATATTACATGCTAAGGGA	
BMPR1B-SINE-RIP9	F: TCAATGAAACAAGATGGCACT	R: ATCGGCTATATCATGAACCCT	
BMPR1B-SINE-RIP10	F: CCTAGATATTGAACGACCATGC	R: TGCCATTTTACTGACTTGGTG	
BMPR1B-LINE-RIP11	F: CCTGACCCTCTTTCATTACACC	R: AGTTCCTGTGCTCTTAACACC	
BMPR1B-ERV-RIP12	F: ACTTTCATTCAATTACAAAGGCTA	R: TCTGAGCGTTGTAACATGACA	
BMPR1B-LINE-RIP13	F: ACCTCACATTAAGAGCCGAGA	R: AATTTCCATGTACTGCGAGTG	
BMPR1B-RIP9	CgacgcgtcgCTTTTGTTGTTGTTGTTGTTGCT	CggaattccgGAGGAGTATTGGGAGTTCCCA	vectors construction
BMPR1B-RIP13	CgacgcgtcgAGCTCTCACACATGCTATGT	CggaattccgAAACTGCTCAGTATACCTTAGAAATCT	
GAPDH	ATCTTCCAGGAGCGAGATCCC	ATGGTTCACGCCCATCACAA	q-PCR
BMPR1B	TACCACCCAACACCCGAGT	ACGCATCTCCTAGCAACCTC	
